# Do investors penalize the firms disclosing higher environmental performance? a cross cultural evidence

**DOI:** 10.1007/s11356-022-19716-8

**Published:** 2022-03-22

**Authors:** Muhammad Azeem Qureshi, Tanveer Ahsan

**Affiliations:** 1 Oslo Business School, Oslo Metropolitan University, 0130 Oslo, Norway; 2 Oslo New University College, Lovisenbergata 13, 0456 Oslo, Norway; 3 Rennes School of Business, 2 Rue Robert D’Arbrissel – CS 76522, 35065 Rennes CEDEX, France

**Keywords:** Environmental disclosure, Climate change, Hofstede cultural dimensions, Value relevance, Social demand, Stakeholders theory, Trade-off theory, C23, G12, G41, L21, Q56, Q58

## Abstract

Climate change discourse integrates 3Ps – people, planet, and profit. However, we do not find any empirical study that integrates 3Ps. Therefore, using a large global panel dataset from 46 countries, this study intends to fill this gap by providing empirical evidence about investors’ value proposition of corporate climate performance in different cultural environments. The results show that Hofstede’s cultural dimensions affect corporate climate performance and its market pricing. We find that in cultures with high power distance, low individualism, high masculinity, high uncertainty avoidance, high long-term orientation, and high restraint, the investors generally penalize the firms disclosing higher environmental performance. Strangely enough, corporate waste production is universally value irrelevant. Our results indicate some policy implications.

## Introduction

Recent empirical evidence of investors’ negative value perception of carbon disclosure (Alsaifi et al., 2020) and recent removal of Emmanuel Faber^1^ highlight the tensions between the investors and the firm as well as its top management in dealing simultaneously with the challenges and the trade-offs of pursuing profits and sustainability (ESG) goals (Hahn et al., 2015) to mitigate climate change risk (Hegerl and Cubasch, 1996). Consequently, we raise a question: Do investors penalize the firms disclosing higher environmental performance? The theoretical and empirical works suggest a positive role of ESG disclosure in the effective allocation of capital by investors that should bring market pressure to improve sustainability practices and thus contribute to sustainable development (Mǎnescu, 2011; Qureshi et al., 2020; Zeidan and Spitzeck, 2015). However, we observe that public discourse on environmental issues and ensuing ‘policy-generated environmental imperatives’ could not equally appeal to ‘corporate environmental conscience’ (Broadstock et al., 2018) across different cultures where firms are likely to have different environmental sensitivity in response to different normative and mimetic pressures from the society (Daddi et al., 2020; Jakučionytė-Skodienė and Liobikienė, 2021) as social norms shape environmental behavior (Yu et al., 2019). Even though more and more investors rely on ESG scores (Folqué et al.), yet certain value-relevant ESG attributes are not efficiently reflected in the stock prices (Mǎnescu, 2011). Consequently, we observe a lot of variation in disclosure of different elements of ESG by the firms domiciled in different countries, indicating stakeholders’ different perceptions about value relevance of different ESG endeavors in different settings necessitating to gather insights in a wider systemic cross-country context (Miska et al., 2018) focusing on investors’ value proposition in corporate environmental performance (CEP) that is generally overlooked in the literature. To fill this gap, this study uses the lens of country culture to understand and help resolve the tensions in corporate environmental endeavors in different temporal or spatial frames (Hahn et al., 2015). For this purpose, we propose an innovative integrative 3Ps framework – people, planet, and profit – to investigate how investors (people) value (profit/firm value) corporate environmental performance (planet) in different cultures (people) to provide a global cross-cultural evidence and use it to answer the question raised above.

Some studies established a positive relationship between ESG disclosure and firm value in different settings (Matsumura et al., 2013; Qureshi et al., 2020): A UK study indicates a negative correlation (Alsaifi et al., 2020); a US study concludes value relevance of certain ESG attributes (Mǎnescu, 2011); a recent study concludes value irrelevance of environmental pillar of ESG (Jadoon et al., 2021); and another recent study of Chinese firms finds a positive association of CEP with corporate financial performance (CFP) as well as the firm value of financially non-constrained firms; however, this relationship turns negative for financially constrained firms (Akbar et al., 2021). As such, ESG-value nexus is still inconclusive (Fatemi et al., 2018; Hannah et al., 2020) due to short-term-versus-long-term trade-offs experienced by the firms (Delmas et al., 2015) in different settings wherein culture as a value system (Meadows, 1998; Miska et al., 2018) shapes the practices of individuals as well as institutions of society (G. Hofstede, 2001; Ioannou and Serafeim, 2012; Yu et al., 2019). However, none of the previous studies investigating ESG-value nexus used all six cultural dimensions (Geert Hofstede, 1984; Minkov et al., 2013), but some of the studies used some cultural dimensions (Gallego-Álvarez and Ortas, 2017; Hartmann and Uhlenbruck, 2015; Ho et al., 2012; Husted, 2005; Ioannou and Serafeim, 2012; Miska et al., 2018; Park et al., 2007; Ringov and Zollo, 2007) and only one study uses all six dimensions to investigate corporate environmental reporting (Gallego-Álvarez and Ortas, 2017). To fill this gap, this study uses all six cultural dimensions and contributes a theoretical 3Ps framework to stakeholders’ theory of the literature to investigate sustainability-value nexus. On the empirical side, the study contributes by demonstrating clear differences of the corporate environmental performance (CEP) as well as its valuation in different cultures to suggest a need for multidimensional cultural sensitivity in climate change policy in the corporate arena.

We organize the rest of the study as follows. The “Theoretical framework” section develops the theoretical framework and the hypotheses; the “Data and methodology” section describes the data and the methodology; the “Results and discussion” section presents the results. Finally, the “Conclusions and policy implications” section provides conclusions and policy implications. We furnish references at the end.

## Theoretical framework

Diverse theoretical underpinnings provide explanations to ESG-firm value nexus. The pioneering proposition on ESG-CFP, trade-off theory (Friedman, 1970), argues that a firm’s sole social responsibility is to maximize economic benefits for its shareholders, and any spending on ESG is a useless increase in operating cost and reduction in profitability. Contrarily, proponents of stakeholders theory postulate a firm’s right to operate as a social contract granted by the stakeholders in society (Scherer and Palazzo, 2007) that requires continuous renewal through the firm’s ESG practices (Carroll, 1979). Such a grounding legitimizes meaningful, dynamic, and long-term engagement of other stakeholders besides the shareholders for a firm’s success (Fatemi et al., 2018). Consequently, a good performance on ESG can have much wider implications to improve corporate economic performance (Fujii et al., 2013) and provide a competitive advantage to create ‘shared value’ (Porter and Kramer, 2011), for example, for European firms (Ahsan and Qureshi, 2021; Qureshi et al., 2020) and US firms (Matsumura et al., 2013). As the cultures are socially constructed, therefore, due to different degrees of perceived compatibility between CEP and firm value in cross-cultural and institutional settings (Ramanathan et al., 2018; Usunier et al., 2011), stakeholders find it difficult to assess this long-run value proposition (Broadstock et al., 2019). Hence, this study uses a panel data technique to carry out global analysis using Hofstede’s six cultural dimensions (Geert Hofstede, 1984; Minkov and Hofstede, 2012) to explicate how investors in different cultural contexts view the value proposition in CEP.

### Power distance index (PDI)

Power distance is the degree of existence and acceptance of inequality of power between the people. In a high-PDI society, powerful people higher in the hierarchy use authoritative decision-making and higher information asymmetry to protect perpetually their socio-economic interests (G. Hofstede, 2001). As such, we expect that the firms in high-PDI countries would maintain lower CEP and the investors would penalize the firms that voluntarily disclose higher CEP by paying a lower price for their stocks and develop our first hypothesis as follows.

- ***H1.**** In high-PDI countries, the firms have lower CEP and there is a negative association between CEP and firm value.*

### Individualism vs. collectivism (IDV)

The collectivist, low-IDV societies integrate an individual to extended families and other in-groups with undoubted loyalty and mutual benefit to favor in-group at the cost of out-group (Chan and Cheung, 2012). Therefore, we expect that firms in low-IDV will try to improve firm value even at the expense of the environment and the investors would penalize the firms that voluntarily disclose higher CEP by paying a lower price for their stocks, leading us to the second hypothesis.

- ***H2.**** In low-IDV countries, the firms have lower CEP and there is a negative association between CEP and firm value.*

### Masculinity vs. femininity (MAS)

A high-MAS masculine society is a hierarchical and traditionally patriarchal society where money and achievements are important; people generally contribute to unethical practices, and corporate managers are ethically insensitive and less concerned about the interests of other stakeholders (Blodgett et al., 2001). Therefore, we expect that the firms in high-MAS countries would achieve higher CFP at the cost of lower CEP, and the investors in high-MAS countries would penalize high CEP firms by paying a lower price for their stocks and present our third hypothesis.

- ***H3.**** In high-MAS countries, the firms have lower CEP and there is a negative association between CEP and firm value.*

### Uncertainty avoidance index (UAI)

Typical characterization of high-UAI culture is avoidance of uncertainty and ambiguity through high intolerance for any deviation from formal rules, regulations, and even societal or organizational norms (Ho et al., 2012). Such argument leads to two inferences: One, society and people will be less open to change and innovation, and two, the firms in such highly regulated countries are forced to invest in environmental efforts. Therefore, we expect higher CEP of the firms in high-UAI countries, whereas investors will penalize the firms that voluntarily disclose higher CEP and develop our fourth hypothesis.

- ***H4.**** In high-UAI countries, the firms have higher CEP and there is a negative association between CEP and firm value.*

### Long-term orientation (LTO)

In high-LTO long-term orientation countries, people tend to be thrifty and pragmatic who view adaptation and circumstantial problem solving as a necessity. In contrast, in low-LTO countries, the people are short-term oriented who adhere to traditions and are resistant to change (G. Hofstede, 2001). As such, we expect that firms in a low-LTO society are likely to resist a new type of environmental investment that reduces firms’ current profitability. Consequently, we expect lower CEP in low-LTO countries, and the investors will penalize high CEP firms by paying a lower price for their stocks. Accordingly, we develop our fifth hypothesis as follows.

- ***H5.**** In low-LTO countries, the firms have lower CEP and there is a negative association between CEP and firm value.*

### Indulgence vs. restraint (IVR)

The people in high indulgence (high-IVR) societies enjoy flexible work hours and the balance between work and social life, and material rewards may not easily motivate them. In contrast, the people in high restraint (low-IVR) societies expect material rewards for a job done well, and stricter social and corporate norms restrain them to behave in a rigid and controlled way (Minkov and Hofstede, 2012). Consequently, we expect the firms in low-IVR countries using their strict corporate norms and material reward system will build only those eco-friendly processes that help improve their profitability and investors will penalize the firms that could not simultaneously maintain higher CEP as well as CFP. As such, we suggest the following hypothesis.

- ***H5.**** In low-IVR countries, the firms have higher CEP and there is a negative association between CEP and firm value.*

The following Table 1 provides a synthesis of the theoretical framework and consequent hypotheses.

**Table 1:** Synthesis of the theoretical framework

Cultural dimension	Expected CEP	Expected relationship of CEP with share price
High-PDI	Low	Negative
Low-IDV	Low	Negative
High-MAS	Low	Negative
High-UAI	High	Negative
Low-LTO	Low	Negative
Low-IVR	High	Negative

## Data and methodology

### Data

We use three proxies for our explanatory variable corporate environmental performance (CEP): One, *CO*_2_*R*_*it*_ – natural log of total Co_2_ emission over natural log of total revenues; two, *WTR*_*it*_ – total waste produced over total revenues; and three, *WRW*_*it*_ – total waste recycled over total waste produced. We use Thomson Reuters Eikon to collect our sample dataset based on three criteria. First, we include all those firms that have disclosed their Co_2_ emissions data at least once in the last three years. Second, we include those countries in the sample for which we have Hofstede’s all six cultural dimensions scores available.^2^ Third, we retain only those countries in our sample for which there are at least ten firm-year observations during 2010–2019. After screening, we obtain a rich dataset of 28,502 firm-year observations of 3115 firms from 46 countries. Considering the model specification, we need a one-year lag of our independent variables that leads us to a final sample size of 25,387 firm-year observations of 3098 firms from 46 countries. The sampled firms belong to 11 different sectors.^3^ Table 2 summarizes the dependent, independent, control, and moderating variables along with their measurement proxies, and Table 3 presents the number of sample firms, firm-year observations in each sample country, along with the classification (low or high) of each country on six dimensions of culture. Please note that we classify a country low on one dimension if its score on that dimension is below the median score on that dimension of all the countries in the sample, otherwise high on that dimension.

**Table 2:** Dependent and independent variables, the model name, and the proxy

Variable level	Variable name	Model Name	Proxy
Dependent	Firm value	*P*_*it*_	Share price close as of the fiscal period end date
Explanatory	Co_2_ emissions	*CO*_2_*R*_*it*_	Natural log of total Co_2_ emission over natural log of total revenues
	Total waste	*WTR*_*it*_	Total waste produced over total revenues
	Waste recycling	*WRW*_*it*_	Total waste recycled over total waste produced
Control	Earnings per share	*EPS*_*it*_	Net income before extraordinary items/average shares outstanding
	Book value per share	*BVPS*_*it*_	Total equity/average shares outstanding
	Firm size	*SZ*_*it*_	Natural log of total assets
	Leverage	*LEV*_*it*_	Total debt/total assets
Moderating	Power distance index	*PDI*_*j*_	Dummy 1 for a high median value of power distance index and 0 otherwise
	Individualism	*IDV*_*j*_	Dummy 1 for a high median value of individualism and 0 otherwise
	Masculinity	*MAS*_*j*_	Dummy 1 for a high median value of masculinity and 0 otherwise
	Uncertainty avoidance	*UAI*_*j*_	Dummy 1 for a high median value of uncertainty avoidance and 0 otherwise
	Long-term orientation	*LTO*_*j*_	Dummy 1 for a high median value of long-term orientation and 0 otherwise
	Indulgence	*IVR*_*j*_	Dummy 1 for a high median value of indulgence and 0 otherwise

**Table 3:** Countries included in the study and their classification on six cultural dimensions

Sr #	Country	Firms	Freq	Percent	Cum	Ranking on six cultural dimensions					
						PDI	IDV	MAS	UAI	LTO	IVR
1	Argentina	15	143	0.50	0.50	Low	High	High	High	Low	High
2	Australia	130	1204	4.22	4.73	Low	High	High	Low	Low	High
3	Austria	28	260	0.91	5.64	Low	High	High	High	High	High
4	Belgium	26	233	0.82	6.46	High	High	High	High	High	High
5	Brazil	56	534	1.87	8.33	High	Low	Low	High	Low	High
6	Canada	120	1127	3.95	12.28	Low	High	Low	Low	Low	High
7	Chile	24	229	0.80	13.09	High	Low	Low	High	Low	High
8	China	172	1468	5.15	18.24	High	Low	High	Low	High	Low
9	Colombia	18	173	0.61	18.84	High	Low	High	High	Low	High
10	Denmark	28	263	0.92	19.77	Low	High	Low	Low	Low	High
11	Finland	33	317	1.11	20.88	Low	High	Low	Low	Low	High
12	France	118	1092	3.83	24.71	High	High	Low	High	High	Low
13	Germany	110	1014	3.56	28.27	Low	High	High	Low	High	Low
14	Greece	15	139	0.49	28.76	Low	Low	High	High	High	High
15	Hong Kong	108	1003	3.52	32.27	High	Low	High	Low	High	Low
16	Hungary	3	30	0.11	32.38	Low	High	High	High	High	Low
17	India	56	536	1.88	34.26	High	High	High	Low	High	Low
18	Indonesia	9	82	0.29	34.55	High	Low	Low	Low	High	Low
19	Ireland; Republic of	26	241	0.85	35.39	Low	High	High	Low	Low	High
20	Italy	84	719	2.52	37.92	Low	High	High	High	High	Low
21	Japan	319	3064	10.75	48.67	Low	High	High	High	High	Low
22	Korea; Republic (S. Korea)	71	640	2.25	50.91	Low	Low	Low	High	High	Low
23	Kuwait	4	40	0.14	51.05	High	Low	High	High	Low	Low
24	Luxembourg	13	104	0.36	51.42	Low	High	Low	High	High	High
25	Malaysia	40	375	1.32	52.73	High	Low	Low	Low	Low	High
26	Mexico	32	302	1.06	53.79	High	Low	High	High	Low	High
27	Netherlands	47	387	1.36	55.15	Low	High	Low	Low	High	High
28	New Zealand	26	229	0.80	55.95	Low	High	High	Low	Low	High
29	Norway	43	383	1.34	57.30	Low	High	Low	Low	Low	High
30	Peru	7	68	0.24	57.54	High	Low	Low	High	Low	Low
31	Philippines	14	140	0.49	58.03	High	Low	High	Low	Low	Low
32	Poland	19	175	0.61	58.64	High	High	High	High	Low	Low
33	Portugal	11	107	0.38	59.02	High	Low	Low	High	Low	Low
34	Qatar	4	39	0.14	59.15	High	Low	High	High	Low	Low
35	Russia	22	210	0.74	59.89	High	Low	Low	High	High	Low
36	Saudi Arabia	4	40	0.14	60.03	High	Low	High	High	Low	Low
37	Singapore	32	312	1.09	61.13	High	Low	Low	Low	High	Low
38	Spain	56	494	1.73	62.86	Low	High	Low	High	High	Low
39	Sweden	81	712	2.50	65.36	Low	High	Low	Low	High	High
40	Switzerland	67	621	2.18	67.54	Low	High	High	Low	High	High
41	Taiwan	99	972	3.41	70.95	Low	Low	Low	High	High	High
42	Thailand	31	297	1.04	71.99	High	Low	Low	Low	Low	Low
43	Turkey	38	361	1.27	73.25	High	Low	Low	High	High	High
44	United Arab Emirates	5	50	0.18	73.43	High	Low	High	High	Low	Low
45	United Kingdom	331	2866	10.06	83.49	Low	High	High	Low	High	High
46	United States of America	520	4707	16.51	100.00	Low	High	High	Low	Low	High
	Total	3115	28,502	100.00							

### Methodology

Our final sample is an unbalanced panel dataset that consists of 25,387 firm-year observations with a time dimension of 2010–2019 and a cross-section dimension of 3098 different firms for which we use the fixed effects technique of panel data analysis. We use the price model of Ohlson (1995) as a benchmark model to measure firm value, already used by many researchers for their empirical studies in related fields (Klerk et al., 2015; Qureshi et al., 2020). The model includes current (*EPS*_*it*_) and past (*BVPS*_*it*_) CFP as the fundamental value drivers. Ohlson (1995) postulates firm value as a function of both financial information and non-financial information. Such an integrated grounding of firm value makes Ohlson’s (1995) model relevant for this study that intends to investigate the value relevance of CEP through the lens of country culture. For this purpose, along with financial information (*EPS*_*it*_, *BVPS*_*it*_), our baseline valuation model includes *CEP*_*it*_ as the non-financial information.

1 $${P}_{it}={\beta }_{0}+{\beta }_{1}{EPS}_{it-1}+{\beta }_{2}{BVPS}_{it-1}+{\beta }_{3}{CEP}_{it-1}+{\varepsilon }_{it}$$

where *P*_*it*_ is the stock price, *EPS*_*it-*1_ is the one-year lagged earnings per share, and *BVPS*_*it-*1_ is the one-year lagged book value per share; *CEP*_*it-*1_ is the one-year lag of one of the three different measures of CEP (total Co_2_ emissions per dollar of revenue, total waste per dollar of revenue, and total waste recycled as percentage of total waste) of firm *i* at time *t,* and ${\varepsilon }_{it}$ is the error term for firm $i$ at time $t$*.*

To investigate the moderating impact of a country’s culture on the relationship between stock price and CEP, we extend Eq. (1) as

2 $${P}_{it}={\beta }_{0}+{\beta }_{1}{EPS}_{it-1}+{\beta }_{2}{BVPS}_{it-1}+{\beta }_{3}{CEP}_{it-1}+{\beta }_{4}{CEP}_{it-1}*{CL}_{j}+{\varepsilon }_{it}$$

where *CL*_*j*_ is a dummy variable for one of the six cultural dimensions (1 for high and 0 otherwise), and *CEP*_*it-*1_*CL_*j*_ is the interaction term of one-year lag of one of the three different measures of CEP of firm *i* at time *t* with a dummy variable for one of the six cultural dimensions. Other variables are the same as explained for Eq. (1)*.*

For the robustness test, we extend our empirical model in Eqs. (1) and (2) to control for firm-specific time-varying variables in the following Eqs. (3) and (4).

3 $${P}_{it}={\beta }_{0}+{\beta }_{1}{EPS}_{it-1}+{\beta }_{2}{BVPS}_{it-1}+{\beta }_{3}{SZ}_{it-1}+{\beta }_{4}{LEV}_{it-1}+{\beta }_{5}{CEP}_{it-1}+{\varepsilon }_{it}$$

4 $${P}_{it}={\beta }_{0}+{\beta }_{1}{EPS}_{it-1}+{\beta }_{2}{BVPS}_{it-1}+{\beta }_{3}{SZ}_{it-1}+{\beta }_{4}{LEV}_{it-1}+{\beta }_{5}{CEP}_{it-1}+{\beta }_{6}{CEP}_{it-1}*{CL}_{j}+{\varepsilon }_{it}$$

where *SZ*_*it-*1_ is the one-year lagged natural logarithm of the total assets, and *LEV*_*it-*1_ is the one-year lagged ratio of total debt over total assets of firm *i* at time *t*. Other variables are the same as explained for Eqs. (1) and (2)*.*

### Robustness and endogeneity issues

For the robustness of our analysis, we perform several diagnostics. First, we use Ohlson’s model that is considered as the best model to investigate ESG-value nexus (Qureshi et al., 2020). Second, we use a one-year lag of all independent variables included in our Ohlson’s model to mitigate the endogeneity issue expected due to the bidirectional relationship between firm value and CEP (Vural‐Yavaş, 2020). Third, we calculate the variation inflation factor (VIF) and find a VIF of less than 10 for all of our regression models; therefore, our models are robust for multicollinearity (Ott and Longnecker, 2015). Fourth, we control for firm size and leverage in our regression models, and the results of our main explanatory variables stay the same with minor differences. Fifth, we take opposite values for our dummy cultural variables (1 for low, and 0 otherwise) in our regression models, and the results of our main explanatory variables stay consistent.^4^

## Results and discussion

Below, we present our results for three proxies of CEP: *CO*_2_*R*_*it*_, *WTR*_*it*_, and *WRW*_*it*_. for six different cultural dimensions.

### Descriptive statistics

The summary statistics in Table 4 (panel A) reveal a lower propensity for CEP disclosure. The median values almost equal to their respective mean values for *CO*_2_*R*_*it*_, *WTR*_*it*_, and *WTR*_*it*_ explain that half of the firms in our sample have an average performance on their carbon footprints, waste production, and waste recycling. Furthermore, mean and median values for *SZ*_*it*_ and *LV*_*it*_ explain that almost half of the sampled firms are of average size, whereas half of the sampled firms have debt a little lower than the average debt. Furthermore, mean and median values for *P*_*it*_, *EPS*_*it*_, and *BVPS*_*it*_ explain that half of the sampled firms have far lower than average current and past profitability and command far lower than the average stock price.

**Table 4:** (**A**) Descriptive statistic and (**B**) mean difference of corporate environmental performance by cultural dimension

	Obs	Mean	Median	STD
Panel A				
*P*_*it*_	28,502	29.406	12.639	44.444
*EPS*_*it*_	28,502	1.552	0.612	2.821
*BVPS*_*jt*_	28,502	15.294	7.034	23.295
*SZ*_*it*_	28,502	22.755	22.635	1.709
*LEV*_*it*_	28,502	0.245	0.234	0.164
*CO*_2_*R*_*it*_	17,695	0.564	0.562	0.099
*WTR*_*it*_	12,406	0.499	0.500	0.285
*WRW*_*it*_	9830	0.512	0.519	0.287
Panel B				
Cultural dimension		*CO*_2_*R*_*it*_	*WTR*_*it*_	*WRW*_*it*_
PDI	Low	0.556	0.486	0.477
	High	0.574	0.510	0.546
	*t*-statistic	− 11.855***	− 4.671***	− 11.983***
IDV	Low	0.573	0.513	0.546
	High	0.556	0.481	0.474
	*t*-statistic	11.035***	6.101***	12.419***
MAS	Low	0.572	0.500	0.460
	High	0.551	0.499	0.581
	*t*-statistic	14.421***	0.221	− 21.088***
UAI	Low	0 .560	0.487	0.469
	High	0.567	0.508	0.541
	*t*-statistic	− 4.732***	− 3.993***	− 12.126***
LTO	Low	0.566	0.494	0.471
	High	0.561	0.504	0.553
	*t*-statistic	3.100***	− 1.926**	− 14.267***
IVR	Low	0.571	0.510	0.556
	High	0.557	0.485	0.461
	*t*-statistic	9.512***	5.020***	16.553***

Panel A of the table presents the descriptive statistics. The use of one-year lagged independent variables reduces the number of firm-year observations in regression models. Panel B presents the results of the mean difference *t*-test for corporate climate performance according to six cultural dimensions. The results for PDI and IDV are as postulated, and the results for MAS, UAI, and IVR are against our postulations, whereas results for LTO are mixed

Panel B of Table 4 presents the results of the *t*-test that demonstrate significant mean differences of the three proxies of CEP, supporting our postulate to investigate investors’ value proposition of CEP from a cultural perspective. As expected, the firms in high-PDI, low-IDV countries have significantly lower CEP on the two proxies (*CO*_2_*R*_*it*_ and *WTR*_*it*_), whereas the results for the third proxy (*WRW*_*it*_) indicate higher CEP against our expectation. From the MAS perspective, our results are contrary to the feminist narrative as well as our expectations, wherein the firms in high-MAS have significantly higher CEP. Against our expectations, the firms in high-UAI countries have significantly higher carbon footprints and produce higher waste per dollar revenue as compared to their counterparts in low-UAI countries. However, as expected, the firms in high-UAI countries have a significantly higher waste recycling ratio as compared to their counterparts in low-UAI countries. As expected, the CEP (using CO_2_*R*_*it*_, *WRW*_*it*_ as proxy) of the firms in low-LTO countries is lower than that of their counterparts in high-LTO countries, whereas the results for *WTR*_*it*_ are against our expectations. Finally, against our expectations, the CEP (using CO_2_*R*_*it*_ and *WTR*_*it*_ as proxy) for the firms in low-IVR countries is lower than that of their counterparts in high-IVR countries, whereas the results for *WRW*_*it*_ are in line with our expectations. Some of our results for the three proxies of CEP for different cultural dimensions are in conformity with whereas others are contrary to our expectations as well as the results of earlier studies; please see Table 1 of Miska et al. (2018). This is another reason for us to use the lens of country culture and three different proxies for CEP to investigate the issue at hand. These results indicate the potential role of varied pressure of institutions and society about different elements of corporate environmental efforts. We argue that it is imperative for all stakeholders to understand and internalize the profound impact of our decisions about the sources and sinks of carbon in the environment. We observe that evolving consensus about the irreversibility of environmental damage due to Co_2_ emissions (carbon sources) has made Co_2_ emissions as the most important policy tool to mitigate climate change risk, generally overlooking waste production and recycling (affecting carbon sinks). This is also reflected in the small percentage of firms reporting their performance on Co_2_ emissions, waste production, and recycling,^5^ clearly suggesting insufficient and varied pressure of institutions and society about different elements of CEP in many countries. Grounding on the effective institutional role in corporate sustainability efforts (Hartmann and Uhlenbruck, 2015; Ioannou and Serafeim, 2012), our results advocate the need to develop across the board uniform benchmarks for Co_2_ emissions, waste production, and recycling and ensure effective enforcement through their corporate regulators and governmental agencies in those cultural contexts where CEP is poor. For example, to reduce Co_2_ emissions, the policymakers should focus their attention on high-PDI, low-IDV, low-MAS, high-UAI, low-LTO, and low-IVR countries; whereas for waste reduction, high-PDI, low-IDV, high-UAI, high-LTO, and low-IVR countries; and to improve waste recycling low-PDI, high-IDV, low-MAS, low-UAI, low-LTO, and high-IVR countries should be the focus of attention of policymakers.

### Correlation analysis

Table 5 presents the results of pairwise correlation between stock prices and one-year lagged explanatory variables. The significant negative correlation of *CO*_2_*R*_*it-*1_ with *P*_*it*_ indicates that higher CEP through lower Co_2_ emissions generally contributes positively to the firm value. However, a significant positive correlation of *WTR*_*it-*1_ and a significant negative correlation of *WRW*_*it-*1_ with *P*_*it*_ are against our expectations, and the conflicting results for *CO*_2_*R*_*it-*1_ and *WTR*_*it-*1_, *WRW*_*it-*1_ motivate us to investigate further with the lens of country culture.

**Table 5:** Pairwise correlation

	*P*_*it*_	*EPS*_*it-*1_	*BVPS*_*it-*1_	*SZ*_*it-*1_	*LEV*_*it-*1_	*CO*_2_*R*_*it-*1_	*WTR*_*it-*1_	*WRW*_*it-*1_
*P*_*it*_	1.000							
*EPS*_*it-*1_	0.770*	1.000						
*BVPS*_*jt-*1_	0.701*	0.740*	1.000					
*SZ*_*it-*1_	0.148*	0.207*	0.273*	1.000				
*LEV*_*it-*1_	− 0.022*	− 0.048*	− 0.040*	0.089*	1.000			
*CO*_2_*R*_*it-*1_	− 0.060*	− 0.050*	0.018*	0.225*	0.256*	1.000		
*WTR*_*it-*1_	0.083*	0.050*	0.032*	− 0.024*	− 0.024*	− 0.233*	1.000	
*WRW*_*it-*1_	− 0.052*	− 0.033*	− 0.002	− 0.008	− 0.036*	− 0.036*	− 0.013	1.000

The table presents the results of pairwise correlation between the dependent and one-year lagged explanatory and control variables. *P*_*it*_ is the share price close at the end of the fiscal year. *EPS*_*it-*1_ is the one-year lag of earnings per share. *BVPS*_*it-*1_ is the one-year lag of book value per share. *SZ*_*it-*1_ is the one-year lag of the natural logarithm of total assets. *LEV*_*it-*1_ is the one-year lag of long-term debt to total assets. *CO*_2_*R*_*it-*1_ is the one-year lag of the natural logarithm of total CO_2_ emissions over the natural logarithm of total revenue. *WTR*_*it-*1_ is the one-year lag of total waste produced over total revenue. *WRW*_*it-*1_ is the one-year lag of total waste recycled over total waste produced over total revenue

^*^Significant at the .05 level

### Value proposition of corporate environmental performance disclosure

Table 6 presents the results for overall data of our empirical model (Eqs. (1) and (3)) that includes three measures of CEP one by one (Model 1 to 3) in addition to the financial variables without and with firm-level controls. Our models suggest that investors value the current CFP (*EPS*_*it-*1_) far more than the past CFP (*BVPS*_*it-*1_) of the firm. Furthermore, the inverse relationship of *P*_*it*_ with *CO*_2_*R*_*it-*1_ in Model 1 depicts investors’ strong appreciation of lesser pollutant firms. However, quite intriguingly, the investors do not consider waste produced (*WTR*_*it-*1_) and waste recycling (*WRW*_*it-*1_) of the firms as value relevant (Model 2 and 3). For robustness, we run the model (Eq. (3)) with control variables (*SZ*_*it-*1_, *LEV*_*it-*1_) and our results stay the same. It is quite puzzling to note such a contrasting behavior of the investors toward Co_2_ emissions and waste production and recycling, leading us to postulate that potentially country culture may provide a better explanation for investors’ response to different elements of CEP. For this purpose, we run the model (Eq. (2)) separately for each of the six dimensions of country culture to investigate how investors in different cultures value CEP disclosure. We present and discuss the results as follows.

**Table 6:** The value proposition of corporate environmental performance

	(1)	(2)	(3)	(1)	(2)	(3)
	*P*_*it*_	*P*_*it*_	*P*_*it*_	*P*_*it*_	*P*_*it*_	*P*_*it*_
*EPS*_*it-*1_	2.963***	2.863***	2.863***	3.014***	2.860***	2.822***
	(0.255)	(0.277)	(0.277)	(0.252)	(0.275)	(0.297)
*BVPS*_*it-*1_	0.461***	0.187**	0.187**	0.363***	0.060	0.047
	(0.085)	(0.077)	(0.077)	(0.106)	(0.080)	(0.092)
*SZ*_*it-*1_				7.839***	9.814***	10.066***
				(1.804)	(1.725)	(1.988)
*LEV*_*it-*1_				25.237***	14.091***	17.009***
				(5.006)	(5.483)	(6.246)
*CO*_2_*R*_*it-*1_	− 21.358**			− 29.966***		
	(8.575)			(8.458)		
*WTR*_*it-*1_		0.208			− 0.238	
		(2.107)			(2.058)	
*WRW*_*it-*1_			− 0.843			− 1.260
			(1.684)			(1.684)
Constant	34.273***	27.183***	29.399***	− 145.565***	− 202.466***	− 206.652***
	(5.025)	(1.800)	(1.722)	(40.436)	(40.078)	(1.722)
Obs	14,964	10,498	8352	14,964	10,498	8352
Firms	2599	1941	1581	2599	1941	1581
*R*-squared	0.626	0.658	0.664	0.468	0.241	0.664
*F*-statistics	65.810***	50.780***	39.460***	50.130***	35.990***	28.420***

The table presents the results of regression analysis controlled for time, sector, and country fixed effects to investigate the impact of climate performance on stock prices of the firms operating in 46 countries around the world during the period from 2010 to 2019. *P*_*it*_ is the share price close at the end of the fiscal year. *EPS*_*it-*1_ is the one-year lag of earnings per share. *BVPS*_*it-*1_ is the one-year lag of book value per share. *SZ*_*it-*1_ is the one-year lag of the natural logarithm of total assets. *LEV*_*it*-1_ is the one-year lag of long-term debt to total assets. *CO*_2_*R*_*it-*1_ is the one-year lag of the natural logarithm of total CO_2_ emissions over the natural logarithm of total revenue. *WTR*_*it-*1_ is the one-year lag of total waste produced over total revenue. *WRW*_*it-*1_ is one-year lag of total waste recycled over total waste produced. The use of one-year lagged independent variables reduces the number of firm-year observations in regression models. Standard errors are in parenthesis

^***^p < 0.01

^**^p < 0.05

^*^p < 0.10

#### Value proposition of corporate Co_2_ emissions disclosure in different cultural contexts

The regression results for our empirical model (Eq. (2)) presented in Table 7 depict a significant positive relationship of *P*_*it*_ with an interaction term of *CO*_2_*R*_*it-*1_ and a cultural dummy for high-PDI, low-IDV, high-MAS, high-UAI, and low-IVR, suggesting that investors in these cultural contexts penalize the firms that disclose higher CEP by reducing their Co_2_ emissions per dollar of revenue supporting all our hypotheses for *CO*_2_*R*_*it-*1_ as a proxy for CEP except for H5. The investors in these cultural contexts plausibly hold the shareholders’ theory perspective and believe that corporate environmental engagement is an unnecessary expense that reduces shareholders’ wealth. Furthermore, the result for low-LTO countries is quite puzzling where investors quite counterintuitively appreciate the firms that put in higher efforts to mitigate climate change and pay higher prices for the stocks of the firms that leave lower carbon footprints per dollar of revenue. This result rejects our hypothesis H5 for CEP using *CO*_2_*R*_*it-*1_ as its proxy. Perhaps, investors in low-LTO countries hold stakeholders’ theory perspective and believe that environmentally responsible corporate conduct helps enhance shareholders’ wealth in the long run. Furthermore, it indicates the need for further investigation on this aspect.

**Table 7:** The value proposition of corporate Co_2_ emissions disclosure in different cultural contexts

	High-PDI	Low-IDV	High-MAS	High-UAI	Low-LTO	Low-IVR
	*P*_*it*_	*P*_*it*_	*P*_*it*_	*P*_*it*_	*P*_*it*_	*P*_*it*_
	(1)	(1)	(1)	(1)	(1)	(1)
*EPS*_*it-*1_	2.961***	2.961***	2.960***	2.964***	2.962***	2.964***
	(0.254)	(0.254)	(0.254)	(0.254)	(0.255)	(0.254)
*BVPS*_*it-*1_	0.461***	0.461***	0.459***	0.461***	0.461***	0.462***
	(0.084)	(0.085)	(0.085)	(0.085)	(0.085)	(0.085)
*CO*_*2*_*R*_*it-*1_	− 53.110***	− 46.678***	− 31.664***	− 44.009***	− 1.913	− 46.818***
	(15.671)	(8.368)	(11.838)	(13.429)	(11.501)	(10.072)
D_Culture* CO_*2*_*R*_*it-*1_	64.441***	51.728***	30.601*	48.431***	− 36.476**	54.128***
	(17.405)	(17.375)	(16.189)	(17.566)	(17.512)	(17.097)
Constant	35.734***	34.667***	33.306***	33.150***	34.976***	34.213***
	(5.326)	(5.133)	(4.910)	(5.059)	(5.194)	(5.031)
Obs	14,964	14,964	14,964	14,964	14,964	14,964
Firms	2599	2599	2599	2599	2599	2599
*R*-squared	0.215	0.309	0.478	0.361	0.450	0.299
*F*-statistics	50.04***	50.06***	49.38***	49.79***	49.72***	51.10***

The table presents the results of regression analysis controlled for time, sector, and country fixed effects to investigate the impact of CO_2_ emissions on stock prices of the firms operating in the countries categorized as high and low on Hofstede’s six cultural dimensions during the period from 2010 to 2019. Standard errors are in parenthesis

^***^p < 0.01

^**^p < 0.05

^*^p < 0.10

#### Value proposition of corporate waste production disclosure in different cultural contexts

Table 8 presents the results of regression analysis for the empirical model (Eq. (2)) using *WTR*_*it-*1_ as a proxy for CEP. Surprisingly, the investors in all cultural contexts do not consider corporate waste production as value relevant, supporting Jadoon et al. (2021) but rejecting all our hypotheses for CEP using *WTR*_*it-*1_ as its proxy. A plausible explanation is that, unlike Co_2_ emissions, corporate waste production has not gained desired attention in public discourse and regulatory frameworks. Consequently, weaker policy-generated environmental imperatives have not sensitized the society and the investors about the role of lower waste production to sustain the carbon cycle’s sinks in the environment.

**Table 8:** The value proposition of corporate waste production disclosure in different cultural contexts

	High-PDI	Low-IDV	High-MAS	High-UAI	Low-LTO	Low-IVR
	*P*_*it*_	*P*_*it*_	*P*_*it*_	*P*_*it*_	*P*_*it*_	*P*_*it*_
	(2)	(2)	(2)	(2)	(2)	(2)
*EPS*_*it-*1_	2.863***	2.863***	2.863***	2.864***	2.863***	2.864***
	(0.277)	(0.276)	(0.276)	(0.277)	(0.277)	(0.277)
*BVPS*_*it-*1_	0.189**	0.189**	0.189**	0.189**	0.189**	0.189**
	(0.077)	(0.077)	(0.077)	(0.077)	(0.077)	(0.077)
*WTR*_*it-*1_	0.173	3.310	1.366	2.048	− 0.020	1.678
	(4.320)	(4.458)	(3.166)	(4.710)	(2.390)	(3.985)
D_Culture* WTR_*it-*1_	0.063	− 5.475	− 3.035	− 2.940	0.432	− 2.703
	(4.647)	(4.708)	(3.782)	(5.073)	(4.148)	(4.457)
Constant	27.183***	27.207***	27.239***	27.176***	27.193***	27.222***
	(1.803)	(1.783)	(1.772)	(1.803)	(1.779)	(1.777)
Obs	10,498	10,498	10,498	10,498	10,498	10,498
Firms	1941	1941	1941	1941	1941	1941
*R*-squared	0.658	0.668	0.658	0.662	0.658	0.665
*F*-statistics	38.23***	38.45***	38.16***	38.20***	38.10***	38.44***

The table presents the results of regression analysis controlled for time, sector, and country fixed effects to investigate the impact of waste production on stock prices of the firms operating in the countries categorized as high and low on Hofstede’s six cultural dimensions during the period from 2010 to 2019

Standard errors are in parenthesis

^***^p < 0.01

^**^p < 0.05

^*^p < 0.10

#### Value proposition of corporate waste recycling disclosure in different cultural contexts

The regression results for our empirical model (Eq. (2)) for corporate waste recycling presented in Table 9 depict a significant negative relationship of *P*_*it*_ with the interaction of *WRW*_*it-*1_ with the cultural dummy for high-PDI, high-UAI, and low-IVR, suggesting that investors in these cultural contexts penalize corporate efforts of waste recycling supporting our hypotheses H1, H4, and H6 for *WRW*_*it-*1_ as a proxy for CEP. Perhaps, investors consider waste recycling an unnecessary cost that reduces CFP in line with shareholders’ theory. However, against our hypothesis H5 (*WRW*_*it-*1_ as a proxy for CEP), we find a significant positive relationship of *P*_*it*_ with the interaction of *WRW*_*it-*1_ with the cultural dummy for low-LTO, indicating that investors in low-LTO society appreciate corporate efforts of waste recycling by paying a higher price for the stocks of the firms that recycle more. Plausibly, the investors in low-LTO culture hold stakeholders’ theory perspective and believe that corporate recycling of waste saves not only the cost that improves CFP but also an environmentally responsible corporate conduct that helps enhance shared value in the long run for all stakeholders, including shareholders.

**Table 9:** The value proposition of corporate waste recycling disclosure in different cultural contexts

	High-PDI	Low-IDV	High-MAS	High-UAI	Low-LTO	Low-IVR
	*P*_*it*_	*P*_*it*_	*P*_*it*_	*P*_*it*_	*P*_*it*_	*P*_*it*_
	(3)	(3)	(3)	(3)	(3)	(3)
*EPS*_*it-*1_	2.834***	2.834***	2.834***	2.831***	2.832***	2.827***
	(0.298)	(0.299)	(0.299)	(0.299)	(0.299)	(0.299)
*BVPS*_*it-*1_	0.172**	0.172**	0.173**	0.171**	0.171**	0.173**
	(0.087)	(0.087)	(0.087)	(0.087)	(0.087)	(0.087)
*WRW*_*it-*1_	2.016	− 1.364	− 1.453	2.745	− 3.807*	2.983
	(2.895)	(3.065)	(2.570)	(3.044)	(2.299)	(2.786)
D_Culture* WRW_*it-*1_	− 5.585*	0.985	1.461	− 6.044*	5.541*	− 7.402**
	(3.383)	(3.466)	(3.167)	(3.579)	(3.308)	(3.392)
Constant	29.495***	29.381***	29.333***	29.552***	29.644***	29.633***
	(1.701)	(1.704)	(1.701)	(1.695)	(1.694)	(1.689)
Obs	8352	8352	8352	8352	8352	8352
Firms	1581	1581	1581	1581	1581	1581
*R*-squared	0.672	0.661	0.661	0.667	0.663	0.668
*F*-statistics	29.96***	29.60***	29.60***	29.80***	29.77***	30.40***

The table presents the results of regression analysis controlled for time, sector, and country fixed effects to investigate the impact of waste recycling on stock prices of the firms operating in the countries categorized as high and low on Hofstede’s six cultural dimensions during the period from 2010 to 2019

Standard errors are in parenthesis

^***^p < 0.01

^**^p < 0.05

^*^p < 0.10

To sum up, we find Hofstede’s cultural dimensions a relevant premise to understand corporate climate performance and investigate its value relevance via the pricing behavior of investors that shapes corporate environmental conduct in the future. Our analyses provide us with five interesting results. First, firms generally disclose varying CEP on three different measures of CEP. Some of our results support, while others reject the results of previous studies. Second, investors also demonstrate a varying degree of appreciation of three different measures of CEP. For the overall data, we find strong appreciation by the investors for the firms that disclose lower carbon footprints per dollar revenue; however, strangely enough, they do not consider corporate waste production per dollar revenue and waste recycling ratio as value relevant providing us the motivation to analyze the data from a country culture perspective. Third, the investors in high-PDI, low-IDV, high-MAS, and low-IVR cultures penalize the firms that disclose higher CEP by leaving lower carbon footprints per dollar of revenue and pay a lower price for the stocks of such firms, whereas investors in low-LTO culture appreciate such firms by paying a higher price for their stocks. Fourth, our results for waste production are quite disturbing wherein we find that globally investors do not consider waste production as value relevant. Fifth, the investors in high-PDI, high-UAI, and low-IVR cultures pay a lower price for the stocks of the firms that disclose higher CEP and claim higher recycling of the waste produced by them and penalize such firms, whereas investors in low-LTO culture appreciate such firms by paying a higher price for their stocks. In the following Table 10, we present the synthesis of theory, our global empirical evidence, and how it compares against the results of earlier studies.

**Table 10:** Synthesis of theory and empirical evidence

Cultural dimension	Corporate environmental performance (CEP)				Relationship of CEP with share price					
	CEP	Co_2_	WTR	WRW	Co_2_		WTR		WRW	
	Expected	Observed	Observed	Observed	Expected	Observed	Expected	Observed	Expected	Observed
High-PDI	Low	Low	Low	High	Negative	Negative	Negative	Insignificant	Negative	Negative
Low-IDV	Low	Low	Low	High	Negative	Negative	Negative	Insignificant	Negative	Insignificant
High-MAS	Low	High	Insignificant	High	Negative	Negative	Negative	Insignificant	Negative	Insignificant
High-UAI	High	Low	Low	High	Negative	Negative	Negative	Insignificant	Negative	Negative
Low-LTO	Low	Low	High	Low	Negative	Positive	Negative	Insignificant	Negative	Positive
Low-IVR	High	Low	Low	High	Negative	Negative	Negative	Insignificant	Negative	Negative

## Conclusions and policy implications

We theorize that country culture affects not only the corporate environmental performance (CEP) but also the investors’ valuation of CEP in the marketplace. We introduce an innovative, integrative 3Ps framework, people, planet, and profit, to consider and investigate this postulate that shapes corporate environmental conduct having long-term implications for sustainable development. For this purpose, we use a rich dataset of 28,502 firm-year observations of 3115 firms from 46 countries over 2010–2019. Our results indicate Hofstede’s cultural dimensions as a relevant premise to understand the nexus of CEP and its valuation by investors in the marketplace to develop a policy framework to effectively address the enormity of climate change for sustainable development. Based on our results, we provide three conclusions that have policy implications.

First, we observe Co_2_ emissions at the core of corporate efforts to mitigate climate change risk, a global neglect of waste production and sporadic attention to waste recycling that threatens environmental endowments across the globe. The three different measures of CEP are not efficiently reflected in the stock prices as observed by an earlier study as well (Mǎnescu, 2011), highlighting the need for the institutions and the policymakers to equitably consider different elements of carbon sources and sinks in developing and implementing sustainable development goals as well as climate change mitigation policy frameworks in the corporate arena. To adopt a holistic policy in the corporate arena to mitigate climate change, we explain policy implications from a social demand perspective. The Co_2_ emissions have been at the center stage of wider public discourse integrating 3Ps creating awareness and sensitivity that created and reinforced social demand internalizing the need for low carbon footprints among all stakeholders. Consequently, active social demand in socio-political circles initiated and later increased regulations by the policymakers. The firms improved their value chain process not only to make them environmentally friendly to comply with the regulatory frameworks set out for Co_2_ emissions but also to make them economically value additive creating a win–win-win solution for 3Ps. However, the policymakers and the society ignored waste and recycling, seriously damaging the sinks of carbon in the natural endowments. We advocate multidimensional policy frameworks that prioritize weaker elements of CEP. For example, investment in technological advancement and innovation should provide economic rationale to pursue cleaner production that reduces the waste and recycles more to help sustain carbon sinks in the natural endowments along with reducing Co_2_ emissions because studies show the potential of sustainable innovations to generate higher future revenues and potential to improve firm value (Zeidan and Spitzeck, 2015). Second, despite the increasing reliance of investors on ESG for firm valuation (Folqué et al., 2021; Qureshi et al., 2020), our results demonstrate a varying degree of value relevance and appreciation of three different measures of CEP for the overall data as well as in six different cultural dimensions. For the overall data, we find a strong appreciation by the investors for lower carbon footprints per dollar revenue, negating Jadoon et al. (2021). However, strangely enough, they consider corporate waste production and recycling ratio as value irrelevant supporting Jadoon et al. (2021). This highlights the need for aroused attention to waste and recycling integrating 3Ps by developing, informing, and enforcing the benchmarks for the corporate arena to create and sustain awareness and sensitivity among all stakeholders to shape and reinforce social demand that internalizes the need to sustain sinks in the natural endowments. However, such an effort should be considerate of short-term-versus-long-term trade-offs faced by the societies and the firms (Delmas et al., 2015) to understand and internalize the dynamic nature of sustainable development. Third, the investors in many cultural contexts penalize the firms that disclose higher CEP (lower Co_2_ emissions and higher waste recycling) by paying a lower price for the stocks of such firms. The policy implication of this conclusion is to incentivize capital allocation to environmentally friendly firms in the capital market of these countries. Overall, we conclude that an integrated societal, institutional, and market pressure that should be equitably considerate of corporate Co_2_ emissions, waste production, and waste recycling should improve corporate endeavors in different cultural contexts to mitigate climate change risk. Our results are limited to the firms declaring Co_2_ emissions at least once during the last three years (only 7.5% of all the listed firms), suggesting not only the limitation of the study but also the enormity of the challenge of climate change mitigation in corporate arena.

## Data Availability

The data that support the findings of this study are available from Thomson Reuters, but restrictions apply to the availability of these data, which were used under license for the current study, and so are not publicly available. Data are however available from the authors upon reasonable request and with permission of Thomson Reuters.

## Footnotes

1 The fall from favor of Danone’s purpose-driven chief | Financial Times (ft.com); Danone: a case study in the pitfalls of purpose | Financial Times (ft.com).

2 We downloaded Hofstede cultural dimensions data from https://data.world/adamhelsinger/geerthofstedeculturaldimension accessed on 18.05.2020.

3 Communication services, consumer discretionary, consumer staples, energy, financials, health care, industrials, information technology, materials, real estate, utilities.

4 To save the space, we do not report the results of robustness tests. Upon request, we will provide the results.

5 Less than 7.5%, 5.2%, and 4.2% in Eikon database accessed on 20.05.2020.

## References

[CR1] Ahsan T, Qureshi MA (2021). The nexus between policy uncertainty, sustainability disclosure and firm performance. Appl Econ.

[CR2] Akbar, A., Jiang, X., Qureshi, M. A., & Akbar, M. 2021. Does corporate environmental investment impede financial performance of Chinese enterprises? The moderating role of financial constraints. *Environmental Science and Pollution Research*. 10.1007/s11356-021-14736-2 10.1007/s11356-021-14736-2 34101121

[CR3] Alsaifi K, Elnahass M, Salama A (2020). Market responses to firms’ voluntary carbon disclosure: empirical evidence from the United Kingdom. J Clean Prod.

[CR4] Blodgett JG, Lu L-C, Rose GM, Vitell SJ (2001). Ethical sensitivity to stakeholder interests: a cross-cultural comparison. J Acad Mark Sci.

[CR5] Broadstock DC, Collins A, Hunt LC, Vergos K (2018). Voluntary disclosure, greenhouse gas emissions and business performance: assessing the first decade of reporting. Br Account Rev.

[CR6] Broadstock DC, Matousek R, Meyer M, Tzeremes NG (2019). Does corporate social responsibility impact firms’ innovation capacity?. The Indirect Link between Environmental & Social Governance Implementation and Innovation Performance Journal of Business Research.

[CR7] Carroll AB (1979). A three-dimensional conceptual model of corporate performance. Acad Manag Rev.

[CR8] Chan AWH, Cheung HY (2012). Cultural dimensions, ethical sensitivity, and corporate governance. J Bus Ethics.

[CR9] Daddi T, Bleischwitz R, Todaro NM, Gusmerotti NM, De Giacomo MR (2020). The influence of institutional pressures on climate mitigation and adaptation strategies. J Clean Prod.

[CR10] De Klerk M, de Villiers C, Van Staden C (2015). The influence of corporate social responsibility disclosure on share prices: evidence from the United Kingdom. Pac Account Rev.

[CR11] Delmas MA, Nairn-Birch N, Lim J (2015). Dynamics of environmental and financial performance: the case of greenhouse gas emissions. Organ Environ.

[CR12] Fatemi A, Glaum M, Kaiser S (2018). ESG performance and firm value: the moderating role of disclosure. Glob Financ J.

[CR13] Folqué, M., Escrig-Olmedo, E., & Corzo Santamaría, T. 2021. Sustainable development and financial system: integrating ESG risks through sustainable investment strategies in a climate change context. *Sustainable Development, n/a*(n/a). 10.1002/sd.2181

[CR14] Friedman, M. 1970. A theoretical framework for monetary analysis. *journal of Political Economy, 78*(2), 193–238.

[CR15] Fujii H, Iwata K, Kaneko S, Managi S (2013). Corporate environmental and economic performance of Japanese manufacturing firms: empirical study for sustainable development. Bus Strateg Environ.

[CR16] Gallego-Álvarez PI, Ortas PE (2017). Corporate environmental sustainability reporting in the context of national cultures: a quantile regression approach. Int Bus Rev.

[CR17] Hahn T, Pinkse J, Preuss L, Figge F (2015). Tensions in corporate sustainability: towards an integrative framework. J Bus Ethics.

[CR18] Hannah, S. T., Sayari, N., Harris, F. H. d., & Cain, C. L. 2020. The direct and moderating effects of endogenous corporate social responsibility on firm valuation: theoretical and empirical evidence from the global financial crisis. *Journal of Management Studies, n/a*(n/a). 10.1111/joms.12586

[CR19] Hartmann J, Uhlenbruck K (2015). National institutional antecedents to corporate environmental performance. J World Bus.

[CR20] Hegerl GC, Cubasch U (1996). Greenhouse gas induced climate change. Environ Sci Pollut Res.

[CR21] Ho FN, Wang H-MD, Vitell SJ (2012). A global analysis of corporate social performance: the effects of cultural and geographic environments. J Bus Ethics.

[CR22] Hofstede G (1984). Cultural dimensions in management and planning. Asia Pacific Journal of Management.

[CR23] Hofstede G (2001). Culture’s consequences: comparing values, behaviors, institutions, and organizations across nations.

[CR24] Husted BW (2005). Culture and ecology: a cross-national study of the determinants of environmental sustainability. MIR Management International Review.

[CR25] Ioannou I, Serafeim G (2012). What drives corporate social performance? The role of nation-level institutions. J Int Bus Stud.

[CR26] Jadoon IA, Ali A, Ayub U, Tahir M, Mumtaz R (2021). The impact of sustainability reporting quality on the value relevance of corporate sustainability performance. Sustain Dev.

[CR27] Jakučionytė-Skodienė M, Liobikienė G (2021). Climate change concern, personal responsibility and actions related to climate change mitigation in EU countries: cross-cultural analysis. J Clean Prod.

[CR28] Mǎnescu C (2011). Stock returns in relation to environmental, social and governance performance: mispricing or compensation for risk?. Sustain Dev.

[CR29] Matsumura EM, Prakash R, Vera-Muñoz SC (2013). Firm-value effects of carbon emissions and carbon disclosures. Account Rev.

[CR30] Meadows DH (1998) Indicators and Information Systems for Sustainable Development. https://donellameadows.org/archives/indicators-and-information-systems-for-sustainable-development/

[CR31] Minkov M, Blagoev V, Hofstede G (2013). The boundaries of culture: do questions about societal norms reveal cultural differences?. J Cross Cult Psychol.

[CR32] Minkov M, Hofstede G (2012). Hofstede’s fifth dimension: new evidence from the world values survey. J Cross Cult Psychol.

[CR33] Miralles-Quiros M, d. M., Miralles-Quiros, J. L., & Arraiano, I. G.  (2017). Sustainable development, sustainability leadership and firm valuation: differences across Europe. Bus Strateg Environ.

[CR34] Miska C, Szőcs I, Schiffinger M (2018). Culture’s effects on corporate sustainability practices: a multi-domain and multi-level view. J World Bus.

[CR35] Ohlson JA (1995). Earnings, book values, and dividends in equity valuation. Contemp Account Res.

[CR36] Ott LR, Longnecker MT (2015) An introduction to statistical methods and data analysis (Vol. 7th edition). Cengage Learning, 20 Channel Center Street, Boston, MA

[CR37] Park H, Russell C, Lee J (2007) National culture and environmental sustainability: a cross-national analysis. Journal of Economics and Finance 31(1):104–121. 10.1007/BF02751516

[CR38] Porter ME, Kramer MR (2011) The big idea: creating shared value. Harv Bus Rev 1(2):62–77

[CR39] Qureshi MA, Kirkerud S, Theresa K, Ahsan T (2020) The impact of sustainability (environmental, social, and governance) disclosure and board diversity on firm value: the moderating role of industry sensitivity. Bus Strateg Environ 29(3):1199–1214. 10.1002/bse.2427

[CR40] Ramanathan R, Ramanathan U, Bentley Y (2018) The debate on flexibility of environmental regulations, innovation capabilities and financial performance – a novel use of DEA. Omega 75:131–138. 10.1016/j.omega.2017.02.006

[CR41] Ringov D, Zollo M (2007) The impact of national culture on corporate social performance. Corporate Governance: the International Journal of Business in Society 7(4):476–485. 10.1108/14720700710820551

[CR42] Scherer AG, Palazzo G (2007) Toward a political conception of corporate responsibility: business and society seen from a Habermasian perspective. Acad Manag Rev 32(4):1096–1120

[CR43] Usunier J-C, Furrer O, Furrer-Perrinjaquet A (2011) The perceived trade-off between corporate social and economic responsibility: a cross-national study. International Journal of Cross Cultural Management 11(3):279–302. 10.1177/1470595811413102

[CR44] Vural‐Yavaş, Ç. 2020. Economic policy uncertainty, stakeholder engagement, and environmental, social, and governance practices: the moderating effect of competition. *Corporate Social Responsibility and Environmental Management, n/a*(n/a), n/a. 10.1002/csr.2034

[CR45] Yu T-K, Chang Y-J, Chang IC, Yu T-Y (2019) A pro-environmental behavior model for investigating the roles of social norm, risk perception, and place attachment on adaptation strategies of climate change. Environ Sci Pollut Res 26(24):25178–25189. 10.1007/s11356-019-05806-7 10.1007/s11356-019-05806-7 31256407

[CR46] Zeidan R, Spitzeck H (2015) The sustainability delta: considering sustainability opportunities in firm valuation. Sustain Dev 23(6):329–342. 10.1002/sd.1594

